# Piezosurgical partial ostectomy of the incisive bone for an ossifying fibroma removal in a 4‐year‐old Warmblood gelding

**DOI:** 10.1002/evj.70178

**Published:** 2026-04-26

**Authors:** G. Forni, M. E. Falomo, I. Casalini, M. Isola

**Affiliations:** ^1^ Department of Animal Medicine, Productions and Health University of Padova Legnaro Italy; ^2^ Private Practitioner Florence Italy

**Keywords:** horse, oral surgery, ossifying fibroma, piezosurgery, premaxillectomy

## Abstract

**Background:**

Ossifying fibromas are uncommon in horses and complete surgical excision with premaxillectomy, maxillectomy or mandibulectomy is recommended. Piezosurgery has been previously used in equines only in one study.

**Objective:**

To report a case of ossifying fibroma treated with piezosurgery and to describe its follow‐up.

**Study Design:**

Clinical case report.

**Methods:**

A 4‐year‐old Zangersheide gelding was presented for a mass occupying the space of tooth 202. Histology confirmed the diagnosis of ossifying fibroma, which was removed by resection of soft tissues and a slice of the premaxilla with a piezotome. First intention closure was achieved with a U‐plasty between the soft palate and the gingiva. Afterwards, histology confirmed complete removal of the mass.

**Results:**

At 5 months, the horse did not show signs of recurrence. Surgery was easy to perform with the piezotome and the cosmetic result was excellent.

**Conclusion:**

Piezosurgery is a feasible technique for oral surgeries in horses, although more prospective studies are required.

## 1 INTRODUCTION

Tumours of the oral cavity are rare in horses^1^; however, ossifying fibromas are among the most common, particularly in young animals.^2^ These benign but locally invasive fibrous neoplasms affect several species, including humans.^3^, ^4^, ^5^ As they typically occur in the rostral mandible or premaxilla, complete surgical excision is the treatment of choice in both humans and horses, and rostral resections of the mandible, premaxilla, or maxilla have been previously performed to remove these tumours.^2^, ^6^, ^7^, ^8^

Piezoelectric surgery was first introduced in 1988^9^ and is now widely used in human medicine, especially in dentistry and craniofacial procedures.^10^, ^11^ The piezotome works using an electrical current that moves the ceramic crystals that it contains, leading to an ultrasonic oscillation of its vibrating tip. This mechanism enables it to selectively cut through the bony tissue.^10^ Studies in human surgery have underlined how piezosurgery, compared to conventional bone cutting devices, reduces cellular damage on bony tissue,^12^ consequently improving healing and reducing postoperative pain.^13^ Thanks to these features, it showed particular benefits in the field of otolaryngologic oncologic resections.^14^ The use of this kind of device is gaining interest also in veterinary medicine, especially in oncologic surgeries in small animals.^15^, ^16^ Only one previous paper assessed the feasibility of the use of a piezotome for osteotomy in horses, in particular for paranasal sinuses, with excellent results, yet acknowledging longer surgical time.^17^ The aim of this paper is to report a case of ossifying fibroma in the premaxilla of a gelding, which was successfully removed by a piezosurgical partial ostectomy of the incisive bone.

## 2 CASE DETAILS

### 2.1 Case history and presentation

A 490‐kg, 4‐year‐old Zangersheide gelding was referred to the veterinary teaching hospital of the University of Padova for further assessment of an oral mass that had been detected by the referring veterinarian during a routine dental examination (Figure 1). The horse had been purchased from abroad 2 weeks before, but no information regarding its health conditions was available. An oral examination had been requested as part of a routine health check for newly purchased horses, and the owners had not noticed any difficulties in grasping hay or mastication.

**FIGURE 1:**
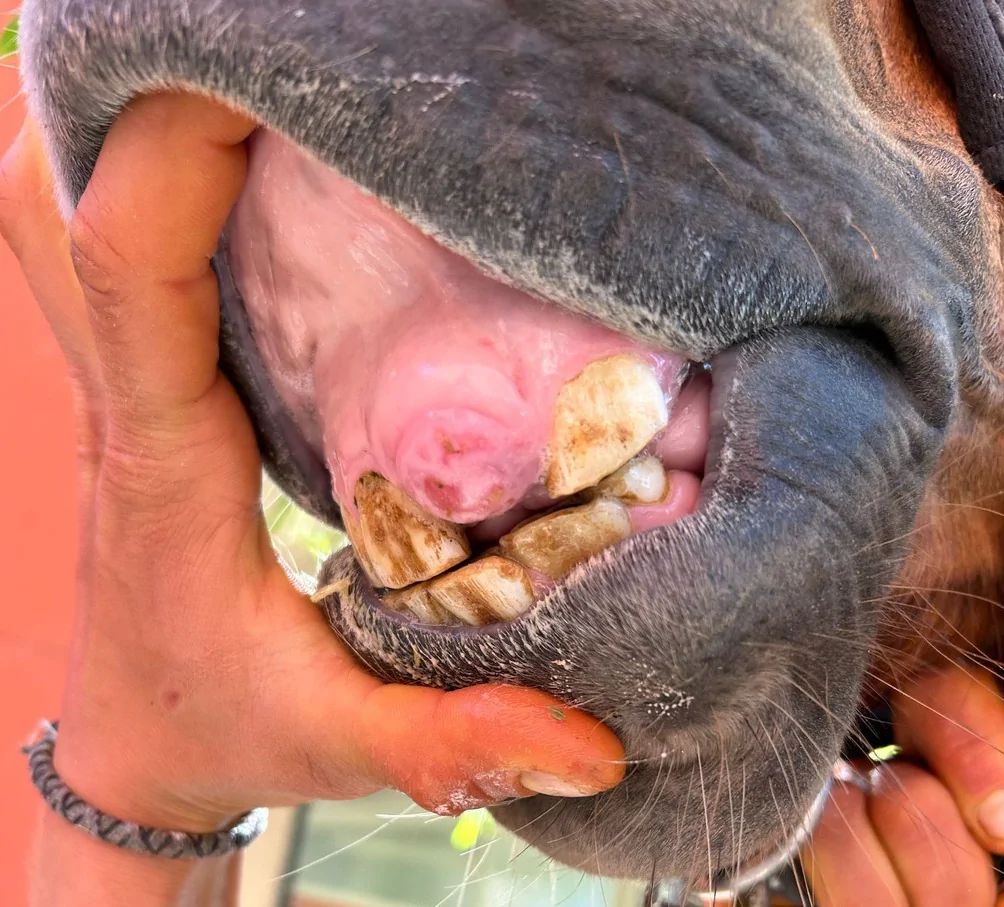
The ossifying fibroma at first examination.

At presentation, the gelding was in good body condition, heart rate (32 beats/min), respiratory rate (12 breaths/min) and rectal temperature (37.2°C) were within normal ranges. Oral examination revealed a mass occupying the space of the intermediate maxillary incisive tooth (202 Triadan nomenclature). The neoplasm appeared to be part of the gingiva, was irregularly rounded, measuring 3 × 3 cm, hard and firm. The surface was ulcerated and small protruding fragments of hard calcified tissue (2–3 mm‐long) were visible. Radiological examination revealed absence of the tooth 202, but no other anomalies were visible. A biopsy was performed under sedation with detomidine (0.02 mg/kg IV) after an infraorbital nerve block of the left nerve was made with 10 mL of 2% lidocaine. A 5 mm diameter punch was used to obtain a sample from the centre of the mass which was immediately fixed in 10% formalin for histological examination.

The specimen showed proliferation of spindle to stellate mesenchymal cells embedded in abundant fibrous stroma. The cells measured approximately 10–15 μm, with variably distinct cytoplasmatic borders, a high nuclear‐to‐cytoplasmic ratio and scant eosinophilic cytoplasm. The nuclei were oval, with condensed chromatin and inconspicuous nucleoli. Mild anisocytosis and anisokaryosis were present. No mitotic figures were identified in 10 high‐power fields. Bone trabeculae lined by variably plump osteoblasts were observed. The overlying epithelium was hyperplastic, displaying an irregular and anastomosing rete ridge pattern. These findings led to the diagnosis of ossifying fibroma; therefore, considering the relatively small size of the tumour, en‐bloc surgical removal was recommended.

### 2.2 Surgical procedure and anaesthetic management

Haematology and blood biochemistry were within normal ranges. Due to the horse's temperament, it was decided to perform the surgery under general anaesthesia. The gelding was fasted for 8 h prior to surgery. Thirty minutes before induction, the horse received penicillin (MSD Animal Health) (22,000 IU/kg IM) and gentamicin (Fatro) (6.6 mg/kg IV) and flunixin meglumine (Equality) (1.1 mg/kg IV). The horse was premedicated with detomidine (Acme) (0.02 mg/kg IV), induction was achieved with ketamine (2.2 mg/kg IV) and diazepam (0.02 mg/kg IV), and after orotracheal intubation, anaesthesia was maintained with isoflurane in air and oxygen. The horse was placed in right lateral recumbency and the upper lip was secured to the nose with a Backhaus clamp. A left infraorbital nerve block was performed using 10 mL of 2% lidocaine (Ecuphar). Surgical scrub of the gingiva was made with a 0.05% chlorhexidine solution and sterile saline.

First, an elliptical incision with a scalpel blade was made on the gingiva and soft palate keeping a 5 mm distance around the edges of the mass, then it was detached from the underlying bone and removed. Subsequently, a partial ostectomy of the incisive bone, parallel to its oral margin, of approximately 6 mm thickness was performed using a 5‐teeth saw tip for the piezosurgery device (Surgysonic II, Esacrom©) (Figure 2). The decision was based on the macroscopical appearance of the bone and on the feeling of the surgeon during the incision. The removed portion of incisive bone was sent for histological examination and assessment of margins.

**FIGURE 2:**
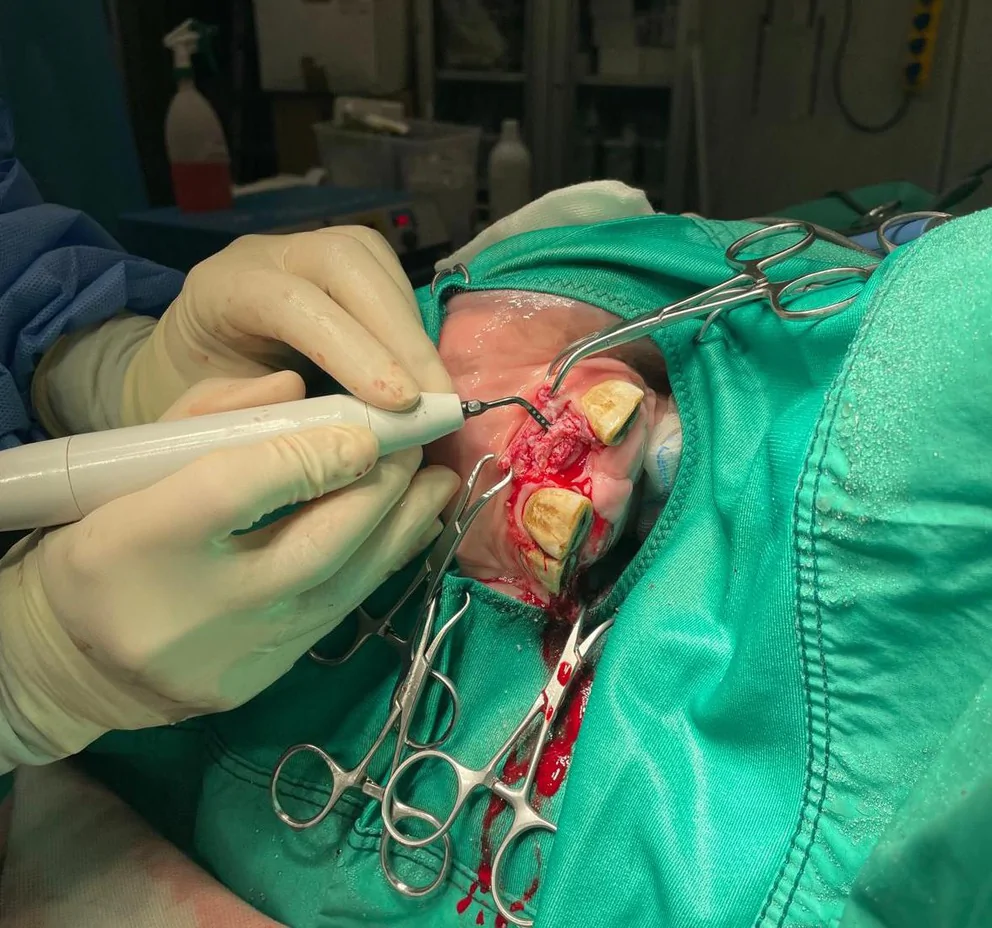
During surgery, while piezosurgical cutting of the incisive bone was made.

Intraoperative bleeding was minimal, and the ostectomy surface was smooth and regular. The cut surface and margins were further refined using a diamond sphere tip (Surgysonic II, Esacrom©). To cover the exposed incisive bone, a U‐plasty was performed by advancing the mucosa of the soft palate cranially, and closure was achieved using a 2–0 monofilament absorbable suture, with simple interrupted stitches. Recovery from anaesthesia was smooth and uneventful (Figure 3).

**FIGURE 3:**
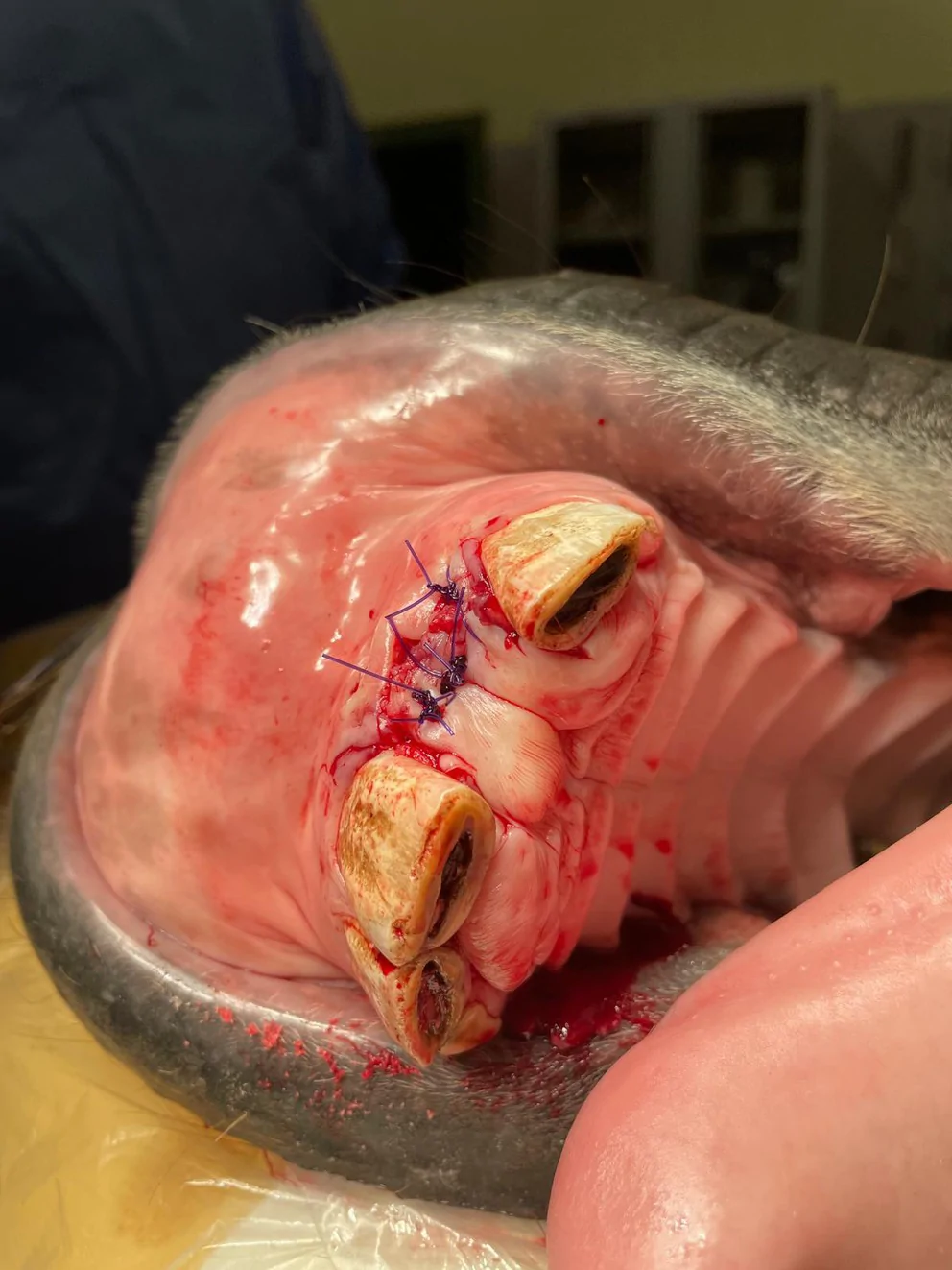
The suture between soft palate and gingiva.

### 2.3 Post‐operative care and outcome

Antibiotics (procaine penicillin 22,000 IU/kg IM q12h and gentamicin 6.6 mg/kg q24h) and non‐steroidal antiinflammatories (flunixin meglumine 1.1 mg/kg q12h) were continued post‐operatively for 3 days. Hay was reintroduced 6 h post‐operatively and the horse appeared comfortable during feeding and mastication. Clinical evaluations were within normal ranges throughout the hospitalisation period and the horse was discharged after 5 days with the recommendation to avoid grains for a further 10 days.

Histological results revealed complete removal of the neoplasia, with margins of the removed mass and bone margins free from neoplastic cells. The horse was re‐examined by the referring veterinarian once a week for 2 weeks and at 2 and 5 months after surgery. Primary wound closure by first intention healing was achieved at 2 weeks post‐operatively. Then, the horse progressively returned to training and was able to compete at its previous level 2 months after surgery (Figure 4).

**FIGURE 4:**
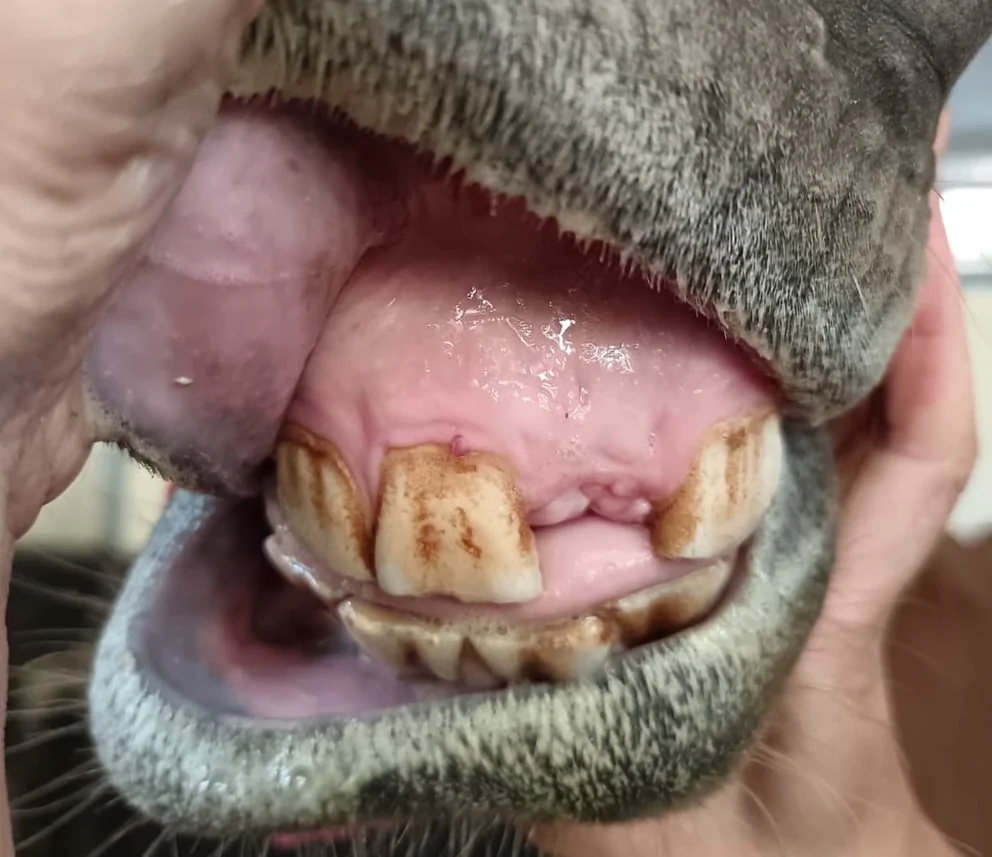
Appearance of the surgical site at 5 months' follow‐up.

## 3 DISCUSSION

This is the first report of piezosurgery being used to perform a partial ostectomy of the incisive bone in a horse. Premaxillar, maxillar and mandibular resections have been previously described in horses mainly through case reports.^6^, ^7^, ^8^, ^18^, ^19^ The main reasons to perform these types of surgeries are tumours affecting the area, as reported also in small animals, in which these procedures are more commonly executed.^16^ In equines, the most commonly encountered tumour is the juvenile ossifying fibroma, which is more likely to affect the mandible than the premaxilla; therefore, mandibulectomies are performed more frequently than premaxillectomies and maxillectomies.^20^, ^21^ Both mandibulectomies and maxillectomies have been performed under standing sedation,^20^, ^21^ which would have been preferable in the present case, both to reduce possible anaesthetic risks and costs, also given the size of the lesion, but the temperament of the horse led to the decision for general anaesthesia to avoid risk for the personnel involved in the procedure.

Ossifying fibromas affect both equines and humans, and are typically encountered in young subjects, although they can occur in adults.^7^ Also, in both species complete surgical excision has been considered to be curative, whereas cases of incomplete removal were associated with high recurrence rates.^22^, ^23^ Nevertheless, since no signs of malignancy are usually present in relapses in humans and re‐excisions are often sufficient, conservative removal is commonly recommended to avoid extensive surgeries.^22^, ^24^ However, in equines, a previous study underlined how incomplete surgical removal was sufficient to avoid recurrence at long‐term follow‐up in some cases, supporting less aggressive surgical approaches, reducing possible complications.^21^ In the present case, histology confirmed complete removal of neoplastic tissue; in fact, no signs of recurrence were present at the 5‐month follow‐up, and it is not expected to occur. The small size of the neoplasm was a favourable feature that allowed its complete removal avoiding a larger ostectomy; in fact, only a 6 mm thick slice was removed from the rostral margin of the premaxilla. This enabled the preservation of the nearby teeth improving the cosmetic result and ensuring that the horse was be able to prehend all types of food, including short grass, which cannot be done when a row of incisors is removed.^23^ This suggests, as in other types of tumours, that early intervention is recommended to avoid extremely aggressive surgeries that may compromise the horse's quality of life. Moreover, more invasive surgeries at the level of the premaxilla and maxilla are at major risk of damaging the major palatine artery.^6^ Whereas in the mandible, with larger ostectomies there is a risk of loss of the symphysis and subsequent instability of the mandible, requiring reconstruction of the joint to avoid long‐term complications such as malocclusion, temporo‐mandibular joint osteoarthritis and nonunion, and regain short‐term function.^8^

Overgrowth of the opposite incisor is expected, but this should not interfere with food prehension and can be easily managed with regular odontoplasty.^23^ In the present case, the tooth 202 was completely absent even at radiographic examination, which is a characteristic feature of permanent hypodontia, likely caused by agenesis, an extremely rare condition in horses.^25^ It is unlikely that the absence of the tooth was related to the development of the neoplasm since histology confirmed that there was no deep involvement of the incisive bone.

Intra‐oral surgical procedures are considered contaminated, since the wide number of species of bacteria that inhabit the oral cavity makes achieving aseptic conditions nearly impossible.^26^ Therefore, in the present case it was decided to administer antibiotic therapy as a prophylactic treatment.^27^

It is worth mentioning that medical treatments have also been reported to treat ossifying fibromas; in fact, radiotherapy and chemotherapy have been used either as a sole treatment or in association with surgery.^28^, ^29^, ^30^ However, given the scarce availability, costs, possible toxicity, and recurrence rate related to these treatments, surgery alone was performed in the present case.

The use of piezosurgery in the present case allowed for precise bone cutting while avoiding damage to the surrounding soft tissues. It proved to be effortless to perform the ostectomy and subsequently smoothing the sharp bony edges that may have caused discomfort, by simply changing the handpiece tip from a 5‐teeth saw to a diamond sphere. These advantages were expected due to the selective action of piezosurgical vibrations on mineralised tissue.^11^, ^31^ Bleeding at the ostectomy site was minimal, which facilitated closure of the overlying soft tissues and likely prevented the wound from swelling in the post‐operative period. In fact, the piezotome produces a cavitation effect that enhances haemostasis,^32^ and also has an antibacterial effect^10^ that has been assessed in equine medicine too, in the treatment of a case of septic arthritis.^33^

Despite the relatively low experience of the surgeon with the device, the piezotome was easily manageable due to its shape, that allows one to hold it as a ‘cutting pen’, as previously reported.^17^ It was not necessary to apply much pressure on the cutting line as long as the tip was kept perpendicular to it. Nevertheless, it is important to acknowledge that tilting the tip while performing the cut may result in either breaking (rare) or slightly bending it, which alters the vibrating mechanism; hence, the machine stops functioning. Other disadvantages related to the piezosurgical device include the initial cost of the machine and tips, which is generally higher than traditional tools. Nevertheless, the same machine can be used for several applications other than osteotomies/ostectomies, such as wound debridement^34^ or articular lavages,^33^ making the purchase more feasible for a referral veterinary hospital. Moreover, both the handpiece and the tips can be reused after sterilisation. Regarding osteotomies, cutting efficiency is reduced compared to conventional devices^11^ and the maximum bone thickness that can be cut is dependent on the length of the tip.

In equine literature, there is only one paper assessing the use of the piezotome as a bone cutting device to perform sinus osteotomies.^17^ Donard and colleagues reported satisfactory results, acknowledging longer surgical time as a possible disadvantage with this technique, as also reported in human orthopaedic surgery,^35^ but in contrast with results in craniofacial surgery, in which no difference in operative times has been noted.^13^ This likely depends on the amount of bone that has to be removed; in fact, the thickness of human cranial bones is lower than those of equines. Although surgical time is expected to increase compared to conventional devices, piezosurgery may improve visualisation by reducing bleeding and enhancing cutting precision. We do not have data regarding the comparison of surgical time with similar surgeries performed with conventional cutting devices, which represents a limitation to the present case report. Another limitation is the short follow‐up available, since recurrences of ossifying fibroma have been reported 2 years post‐operatively.^21^

In conclusion, piezosurgery seems to be a promising and safer alternative to conventional bone cutting devices, although prospective studies are needed to better assess this in equine patients. In cases of oral neoplasia, early intervention is critical to increase chances to perform less extensive surgeries in order to obtain a better quality of life and a better cosmetic result.

## FUNDING INFORMATION

Not applicable.

## CONFLICT OF INTEREST STATEMENT

The authors declare no conflicts of interest.

## AUTHOR CONTRIBUTIONS

**G. Forni:** Conceptualization; methodology; data curation; writing – original draft; writing – review and editing; project administration. **M. E. Falomo:** Methodology; writing – review and editing; data curation. **I. Casalini:** Conceptualization; writing – review and editing. **M. Isola:** Methodology; writing – review and editing; supervision.

## DATA INTEGRITY STATEMENT

G. Forni had full access to all the data in the study and takes responsibility for the integrity of the data and the accuracy of the data analysis.

## ETHICAL ANIMAL RESEARCH

Research ethics committee oversight not required by this journal: retrospective study of clinical records.

## INFORMED CONSENT

The owner provided consent, as part of the veterinary hospital admission consent form, for the use of the horse's data for research.

## Data Availability

The data that support the findings of this study are available upon reasonable request from the corresponding author. Open data sharing exemption granted by the editor.
